# The Antibacterial Activity and Mechanisms of a Mixed Bio-Preservative on the *Bacillus* Stains in Crab Roe Sauce

**DOI:** 10.3390/foods14030525

**Published:** 2025-02-06

**Authors:** Rongrong Yu, Rongxue Sun, Ning Jiang, Bin Zhang, Cheng Wang, Qianyuan Liu, Zhiqiang Li, Xingna Wang

**Affiliations:** 1 Zhejiang Provincial Key Laboratory of Health Risk Factors for Seafood, College of Food and Pharmacy, Zhejiang Ocean University, Zhoushan 316022, China; 19818072135@163.com; 2 Institute of Agricultural Products Processing, Jiangsu Academy of Agricultural Sciences, Nanjing 210014, China; sunrongxue187@163.com (R.S.); wangcheng@jaas.ac.cn (C.W.); liu.qianyuan@foxmail.com (Q.L.); 20080053@jaas.ac.cn (Z.L.); xingnawang.nn@163.com (X.W.); 3 Integrated Scientific Research Base for Preservation, Storage and Processing Technology of Aquatic Products of the Ministry of Agriculture and Rural Affairs, Nanjing 210014, China

**Keywords:** crab roe sauce, specific spoilage bacteria, ε-polylysine hydrochloride, nisin, tea polyphenols

## Abstract

Crab roe sauce (CRS) is prone to spoilage due to microbial contamination. Therefore, this study aimed to investigate the inhibitory effects and mechanisms of a mixed bio-preservative (0.025% ε-polylysine hydrochloride (ε-PL) + 0.01% nisin (NS) + 0.01% tea polyphenols (TPs)) on the specific spoilage bacteria (SSB) in CRS. First, the SSB in CRS were isolated and identified by 16S rRNA sequencing. Two isolates were selected as representative strains based on their enzymatic spoilage potential and spoilage capability in CRS. By comparing the inhibition zones, ε-PL, NS, and TPs were selected from five conventional bio-preservatives (ε-PL, NS, TPs, grape seed extract (GSE), and rosemary extract (RE)) to prepare the mixed bio-preservative. The results showed that the minimum inhibitory concentration (MIC) of the mixed bio-preservative against *Bacillus pumilus* and *Bacillus subtilis* was 56.3 µg/mL. The growth curves and cell viability tests revealed that the mixed bio-preservative reduced the viability of both strains. The conductivity, alkaline phosphatase activity, and nucleic acid and soluble protein leakage indicated that the mixed bio-preservative disrupted the integrity of the cell walls and membranes of the two isolates in a concentration-dependent manner. Scanning electron microscopy further confirmed the damage to the cell membranes of the two isolates by the mixed bio-preservative. Overall, the mixed bio-preservative exhibited excellently inhibitory effects on the SSB and could be a promising method for the preservation of CRS.

## 1. Introduction

Crab roe sauce (CRS) is a novel aquatic seasoning product, with a rich flavor and abundance of nutrients, which could effectively address the seasonal supply and consumption issues of crab products [1]. However, due to its richness in proteins and fats, as well as the influence of multiple factors such as its pH and low salt content, it is highly susceptible to microbial contamination during production and processing. The spoilage microorganisms could expedite protein degradation and lipid oxidation, producing off-odors that render the food inedible. During the heating procedure, CRS is usually mildly pasteurized to maintain its texture as well as its nutritional content. However, this mild process still allows the survival of some spoilage bacteria, e.g., *Bacillus* spp., thus resulting in a short shelf life for the products [2]. Low-temperature storage is a common method to extend the shelf life of CRS. It effectively reduces the activity of microorganisms and enzymes, contributing to the preservation of food quality. However, low-temperature storage can only slow down the growth rate of spoilage bacteria, rather than completely prevent it [3]. During storage, only the specific spoilage bacteria (SSB) multiply extensively, assuming a leading position and ultimately causing product spoilage [4]. The proliferation of SSB can have adverse effects on the quality of CRS. Therefore, preservatives are necessary to provide CRS stored at low temperatures with additional antibacterial and preservative effects.

Chemical preservatives, such as nitrates, nitrites, and benzoates, are commonly used to control spoilage bacteria in foods. Although they can extend the shelf life of foods, they might have adverse effects on human health, and consumers have a low acceptance of added chemicals in food [5]. With the increasing consumer concern about food safety and health, the necessity for bio-preservatives as substitutes for chemical preservatives is growing. Bio-preservatives are a new type of preservative that are green, safe, and efficient, which are primarily derived from animals, plants, and microorganisms. They are characterized by their green safety, high efficiency, and broad-spectrum antimicrobial properties [6], making them a research hotspot in the modern food preservation field. For example, ε-polylysine hydrochloride (ε-PL), which can be obtained from bacterial fermentation, is a novel bio-preservative with broad-spectrum inhibitory properties and thermal stability [7]. It can effectively maintain the quality of aquatic products during the storage process. Currently, it is mainly used for the antibacterial preservation of refrigerated and frozen aquatic products [8]. Nisin (NS), a polypeptide isolated from *Lactococcus lactis* ssp., can effectively inhibit the growth and metabolism of *Listeria monocytogenes* and Gram-positive bacteria [9]. Both these antimicrobial agents are regarded as naturally safe and widely used in preserving fruits, vegetables, pork, and fish [10]. Tea polyphenols (TPs), natural polyphenolic compounds extracted from tea, are regarded as a promising natural preservative because of their excellent antimicrobial and antioxidant activities and are used for preserving fruits and vegetables, grains and oils, and meat and aquatic products [11]. Moreover, previous research showed that adding ascorbic acid, TPs, and NS to CRS and combining with heat sterilization or ultra-high-pressure sterilization treatments could extend the shelf life of CRS under room temperature storage conditions [1].

However, the practical efficacy of natural preservatives is often constrained by their solubility and uniform dispersion, especially their antimicrobial spectrum. When a single natural preservative is used in food, higher concentrations are typically required to achieve a sufficient preservation effect, which may compromise the original flavor of the food [12]. Consequently, combining several different natural preservatives can expand the antimicrobial spectrum and improve antioxidant activity [13]. In this way, mixed bio-preservatives can achieve better antimicrobial and preservation results in food while decreasing the dosage of each preservative required.

As limited studies have been focused on the SSB in CRS, more information is still required on its storage and preservation. Thus, this study aimed to develop a mixed bio-preservative for refrigerated CRS and preliminarily investigated its antibacterial activity and mechanisms against SSB in CRS to provide a theoretical basis for its application in CRS preservation. Firstly, the SSB in refrigerated spoiled CRS were isolated and identified and their spoilage potential was evaluated. Secondly, via in vitro antibacterial experiments, three bio-preservatives with good inhibitory effects on SSB were selected from five common ones and combined. Finally, the minimum inhibitory concentration (MIC) was used as an indicator to preliminarily explore the antibacterial activity and mechanisms of the mixed bio-preservative against SSB in CRS by measuring its impacts on the SSB’s growth curve, cell viability, electrical conductivity, alkaline phosphatase activity, and the leakage of nucleic acids and soluble proteins.

## 2. Materials and Methods

### 2.1. Samples

The pasteurized (95 °C for 20 min) CRS used in this study was provided by Nanjing Huinuo Food Co., Ltd. (Nanjing, China) and was stored at 10 °C. The bio-preservatives employed in this study included NS (≥1000 IU/mg) and ε-PL (with a molecular weight range of 2000–5000 Da), both purchased from Aladdin Bio-Chem Technology Co., Ltd. (Shanghai, China); TPs (98%), purchased from Yuanye Bio-Technology Co., Ltd. (Shanghai, China); and grape seed extract (GSE, 95%) and rosemary extract (RE, 99%), both obtained from Macklin Inc. (Shanghai, China).

### 2.2. Isolation and Identification of SSB

After being stored in a 10 °C refrigerator for 15 days and spoiled, samples of the CRS (10 ± 0.1 g) were transferred to sterile homogenizing bags within a laminar flow bench (SJ-CJ-1D, Suzhou Purification Equipment Co., Ltd., Suzhou, China). After the addition of 90 mL of sterile saline (0.85%), homogenization was performed using a microbiological homogenizer (XO-6D, Xianou Instruments Manufacture Co., Ltd., Nanjing, China) at a slapping speed of 6 times/s and a temperature of 25 ± 1 °C for 2 min. The obtained homogenate was diluted in 10-fold serial dilutions using sterile saline (0.85% NaCl, Sinopharm, Beijing, China). Aliquots of 100 μL from three appropriate dilutions were spread-plated onto Plate Count Agar (PCA, Hopebio Technology Co., Ltd., Qingdao, China). Each dilution was plated in triplicate. The plates were incubated in a constant temperature incubator (SN-HWS-250B, Shangpu Instrument Equipment Co., Ltd., Shanghai, China) at 37 ± 1 °C for 48 h. The purified colonies were selected using the 4 × 4 streak plating method. Distinct colonies of different morphologies were streaked onto PCA plates and aerobically cultivated at 37 ± 1 °C for 48 h. Subsequently, these purified colonies were verified by microscope.

The genomic DNA of the cultivated bacteria was extracted using the TSINGKE Plant DNA Extraction Kit (Tsingke Biotechnology Co., Ltd., Beijing, China) as per the manufacturer’s instructions. The extracted bacterial genomic DNA served as the template for amplification of the target gene fragment using the primers 27F (5′-AGAGTTTGATCMTGGCTCAG-3′) and 1492R (5′-GGTTACCTTGTTACGACTT-3′). The polymerase chain reaction (PCR) was conducted in a 50 μL reaction volume: 45 μL of TSE101 Golden Mix (1×), 2 μL of primer 27F (10 P), 2 μL of primer 1492R (10 P), and 1 μL of DNA template. The PCR protocol entailed an initial denaturation at 98 °C for 2 min, followed by 30 cycles of denaturation at 98 °C for 10 s, annealing at 56 °C for 10 s, and extension at 72 °C for 10 s per kilobase, concluding with a final extension at 72 °C for 5 min and holding at 4 °C. The amplified PCR product was analyzed by agarose gel electrophoresis (2 μL sample + 6 μL bromophenol blue) at 300 V for 12 min, and a gel image was obtained for product quality assessment. Sequencing of the PCR product was outsourced to Nanjing Tsingke Biotechnology Co., Ltd, Nanjing, China. The sequencing results were analyzed using the BLAST function of the National Center for Biotechnology Information (NCBI, https://www.ncbi.nlm.nih.gov/, accessed on 18 December 2024) to compare the 16S rRNA gene sequences against known sequences. According to the species identification protocol, sequences with a similarity of greater than 97% were considered to be of the same species. The isolated strains were suspended in sterile 50% (*v*/*v*) glycerol and stored at −20 °C for future use.

### 2.3. Screening for the Enzyme Production

The determination of specific spoilage-related enzyme activities (amylase, lecithinase, lipase, and protease activities) was conducted based on the method of Xia et al. [14], with minor modifications. An aliquot (5 µL) of the activated bacterial suspension (10^6^ CFU/mL) was spotted onto skim milk agar plates (1 × Tryptic Soy Agar (TSA, Hopebio) + 2% skim milk) and starch agar plates (1 × LB Agar (Hopebio) + 1% starch). After incubation at 37 ± 1 °C for 5 days in a constant temperature incubator, the size of the hydrolysis zone on the skim milk agar plates was used to assess protease activity. Starch agar plates were stained with Lugol’s iodine solution, with the transparent zones around the colonies indicating amylase production. The strains were re-cultured on PCA plates, and individual colonies were streaked onto Tween 80 agar plates (1 × LB Agar + 1% Tween 80 + 0.1 g CaCl_2_) and egg yolk agar plates (1 × LB Agar + 2% egg yolk emulsion) to detect lipase and lecithinase activities, respectively. After incubation at 37 ± 1 °C for 5 days, the formation of white precipitate around the colonies indicated lipase and lecithinase production.

### 2.4. Spoilage Potential Assessment

#### 2.4.1. Preparation and Inoculation of Sterile CRS

Each selected isolate was incubated in Tryptic Soy Broth (TSB) at 37 ± 1 °C in a constant temperature shaker (HZQ-F100, TC-huamei, Taicang, Jiangsu, China) for 20–24 h until 7.0–8.0 log CFU/mL was reached. The suspensions were then centrifuged at 5000 rpm at 4 °C for 10 min using a high-speed centrifuge (H2050R, Xiangyi Laboratory Instrument Development Co., Ltd., Hunan, China) to collect the bacterial pellets. The pellets were washed three times by resuspending in 30 mL of sterile saline and centrifuging at 5000 rpm for 10 min each time, and finally resuspended in 30 mL of sterile saline for further use.

The preparation of sterile CRS and inoculation was conducted according to Song et al. [15] with slight modifications. The CRS was sterilized in an autoclave (LDZM-80L-I, Shanghai ShenAn Medical Instrument Factory, Shanghai, China) at 121 °C for 15 min. Then, each pouch (100 g) of sterile CRS was inoculated with 1 mL of the bacterial suspension to achieve a final concentration of 5.0–6.0 log CFU/g. For the control group (CK), 1 mL of sterile saline was used instead of the bacterial suspension. After inoculation, the CRS was stored at 10 °C for 8 days. For each treatment group (TG), 3 pouches of sauce were randomly selected on days 0, 2, 4, 6, and 8 for analysis.

#### 2.4.2. Microbial Analysis

The total viable count (TVC) was determined following the method of Yang et al. [16]. Briefly, 5 ± 0.1 g of the CRS was placed into a sterile homogenizing bag with 45 mL of sterile saline. The mixture was homogenized using a microbiological homogenizer for 2 min, and then a volume of 100 μL from a series of dilutions was spread-plated onto PCA plates. Afterwards, the PCA plates were incubated at 37 ± 1 °C for 48 h, and then the total number of colonies was enumerated. Colony counts were expressed in colony-forming units per gram (CFU.g^−1^).

#### 2.4.3. Total Volatile Basic Nitrogen (TVB-N) Analysis

The determination of TVB-N was conducted following the method of Prabhakar et al. [17]. The CRS (5 ± 0.001 g) was mixed with 45 mL of distilled water and homogenized for 1 min. Then, 5 mL of the homogenate was combined with an equal volume of 10% trichloroacetic acid (TCA) (*w*/*v*) and filtered using Whatman filter paper (11 μm). A 1 mL volume of each of the filtrates and saturated potassium carbonate were transferred to the outer chamber of a microdiffusion device, which contained an inner ring solution (1 mL). The inner ring solution was prepared by mixing 2% boric acid (92 mL), 4 mL each of a 0.1% methyl red ethanol solution and a 0.1% bromocresol green ethanol solution. After securely sealing the microdiffusion device, it was gently rotated on a tabletop in a circular motion to thoroughly mix the sample and the saturated potassium carbonate solution. The device was incubated at 37 ± 1 °C for 3 h and then cooled to room temperature. The inner ring solution was titrated with 0.002 mol/L sulfuric acid to a purplish-red color. A blank test was conducted simultaneously, replacing the sample filtrate with 10% TCA (1 mL). The TVB-N results were expressed as milligrams per 100 g of sample (mg/100 g).

#### 2.4.4. Yield Factor Calculation of Decay Ability

The spoilage potential of each spoilage strain was quantified using the method described by Huang and Xie [18], with the production factor (Y_TVB-N/CFU_) serving as a metric to evaluate the spoilage capacity of the strains. The calculated results were obtained using the following Equation (1):

(1) $$Y_{\mathrm{TVB}-N/\mathrm{CFU}}\;=\frac{(TVB-N)s-(TVB-N)0}{CFUs-CFU0}$$

(TVB-N)_0_ and (TVB-N)_S_ represent the TVB-N content in the CRS samples at the initial and final points of storage after inoculation with the bacterial suspension, respectively, which were measured in units of mg/100 g. CFU_0_ and CFU_S_ denote the TVC in the CRS samples at the initial and final points of storage, respectively, measured in units of log_10_ CFU/g.

### 2.5. Antimicrobial Activity of the Mixed Bio-Preservative

#### 2.5.1. Diameter of Inhibition Zones of Single Bio-Preservative

On a sterile laminar flow bench, a series of gradient solutions of each bio-preservative (NS, ε-PL, TPs, GSE, and RE) were prepared with sterile distilled water at concentrations of 0.5%, 0.4%, 0.3%, 0.2%, and 0.1% (*w*/*v*). The prepared solutions were employed as the bio-preservatives for the preliminary screening in this experiment. Then, the antimicrobial properties of each bio-preservative were assessed using the well diffusion method [19]. In a sterile laminar flow hood, 100 μL of a bacterial suspension with a concentration of 10^6^ CFU/mL was spread onto the surface of PCA plates. With a sterile cork borer, six 6 mm diameter wells were punched in the plate and the excess agar was removed. A pipette was used to dispense 100 μL of the bio-preservatives at varying concentrations into each well. For the CK, 100 μL of sterile distilled water was used instead. After incubation at 37 ± 1 °C for 24 h, the diameter of the inhibition zones was measured using a vernier caliper via the cross-sectional method. The antimicrobial activity of the bio-preservatives was evaluated based on the size of the inhibition zones. The diameter of the inhibition zones (mm) was calculated as the diameter of the inhibition zones minus 6 mm. The final value was the mean of three parallel measurements ± standard deviation.

#### 2.5.2. Determination of the MIC of the Mixed Bio-Preservative

In this study, the three most effective bio-preservatives identified through the screening were formulated in combination according to the maximum permitted addition levels stipulated in the National Standards of China (GB 2760-2014). An analytical balance was used to accurately weigh 0.25 g of ε-PL, 0.10 g of NS, and 0.10 g of TPs, which were then dissolved in 1 L of sterile distilled water to prepare a mixed bio-preservative solution with a concentration of 0.45 g/L. The MIC of the mixed bio-preservative was determined using the microbroth dilution method [20]. A sterile 96-well plate was prepared, with 100 μL of sterile TSB added to the wells in column 1 to column 12. Column 1 received 100 μL of sterile distilled water as a negative control, and column 12 received 100 μL of a bacterial suspension as a positive control. Column 2 was loaded with 100 μL of the bio-preservative at the specified concentration. The solution was mixed and then 100 μL was transferred to column 3, and this serial dilution was continued up to column 11. After mixing column 11, 100 μL was discarded, ensuring that the concentration of the bio-preservative in each subsequent well was halved. The bacterial suspension was diluted to 10^6^ CFU/mL in sterile TSB broth, and 100 μL was added to wells 2 through 11. Rows 1–3 of the 96-well plate were inoculated with a *Bacillus pumilus* bacterial suspension, and rows 4–6 were inoculated with a *Bacillus subtilis* bacterial suspension. The 96-well plate was then incubated at 37 ± 1 °C for 24 h. Following incubation, the absorbance of each well at 600 nm was measured using a multifunctional microplate reader (Spark, Tecan Austria LLC, Grödig, Austria) and checked by visual observation.

### 2.6. Growth Curves Determination

The impact of the mixed bio-preservative on the growth processes of two SSB was investigated by measuring the absorbance of the bacterial suspensions at 600 nm [21]. The mixed bio-preservative was added to sterile TSB followed by the introduction of bacterial suspensions of *B. pumilus* and *B. subtilis*, each at a concentration of 10^6^ CFU/mL. The final concentrations of the mixed bio-preservative in each group were set to 1/4MIC, 1/2MIC, 1MIC, and 2MIC, while the CK received an equivalent volume of sterile distilled water. The samples were placed in a multifunctional microplate reader and incubated at 37 ± 1 °C for 24 h, with the OD values (OD600) of each bacterial suspension being measured every 2 h.

### 2.7. Determination of Electrical Conductivity

The electrical conductivity was measured according to Xu et al. [22]. Bacterial suspensions, cultured to the logarithmic growth phase (10^6^ CFU/mL), were centrifuged at 4 °C and 5000 rpm for 10 min. The supernatant was discarded, and the bacterial pellets were washed three times with sterile PBS buffer (0.01 M, pH 7.2) and resuspended. The mixed bio-preservative was added to reach final concentrations of 1/2MIC and 1MIC, with sterile distilled water used as the CK. The samples were then incubated in a constant temperature shaker (37 ± 1 °C, 220 rpm) for 12 h. Samples were taken every 2 h, centrifuged at 5000 rpm for 10 min, and the supernatant’s electrical conductivity was measured using a portable multi-parameter analyzer (DZS-708T, Lei Ci Co., Ltd., Shanghai, China).

### 2.8. Determination of Nucleic Acid Leakage

The nucleic acid leakage was measured following the method of Cui et al. [23], with minor modifications. The bacterial supernatant samples were prepared similarly to the procedure described in Section 2.7, and the absorbance of the supernatant was measured at 260 nm with a UV-Vis spectrophotometer (UV-6300, Mapada Instruments Co., Ltd., Shanghai, China).

### 2.9. Determination of the Soluble Protein Content

The bacterial supernatant samples were prepared similarly to the procedure described in Section 2.7, and the supernatants were used to quantify the soluble protein content using a BCA kit (Nanjing Jiancheng Bioengineering Institute, Nanjing, China) [24].

### 2.10. Determination of Alkaline Phosphatase (AKP) Activity

The measurement of AKP activity followed the method of Yuan et al. [25]. The bacterial supernatant samples were prepared similarly to the procedure described in Section 2.7. The AKP content of the supernatant was determined according to the instructions of the AKP kit (Nanjing Jiancheng Bioengineering Institute, Nanjing, China).

### 2.11. Determination of Cell Viability

The changes in cell viability of the two SSB were assessed using a CCK-8 cell viability assay kit (Nanjing Jiancheng Bioengineering Institute, Nanjing, China). Specifically, 100 μL of cell suspension (10⁶ CFU/mL) was first added to each well of a 96-well plate and pre-incubated in an incubator for 24 h (37 °C, 5% CO₂). Subsequently, 100 μL of TSB with the mixed bio-preservative at either 1MIC or 2MIC was added to the TGs, while only 100 μL of TSB was added to the CK. Next, the plate was incubated for another 48 h. Then, 10 μL of the CCK-8 solution was added to each well. Finally, the plate was incubated for 4 h and the absorbance was measured at 450 nm using a multifunctional microplate reader. The cell viability was calculated using the following Equation (2):

Cell viability (%) = (T/C) × 100(2)

T represents the OD value of the cells treated with the mixed bio-preservative at different concentrations at 450 nm, and C represents the OD value of the control group cells at 450 nm.

### 2.12. Scanning Electron Microscope (SEM) Analysis

Surface morphological changes in bacteria following 12 h of treatment with the mixed bio-preservative were observed using SEM and established methods, with modifications [26]. The bacterial samples were prepared similarly to the procedure described in Section 2.7. One milliliter of each bacterial suspension was then transferred to sterilized centrifuge tubes and centrifuged at 5000 rpm for 10 min at 4 °C. The supernatants were discarded, and the bacterial pellets were washed three times with sterile PBS buffer (0.01 M, pH 7.2) and centrifuged again. The pellets were then fixed in 2.5% (*v*/*v*) glutaraldehyde at 4 °C for 12 h. The samples were dehydrated in graded ethanol solutions (75%, 80%, 90%, and 95%) for 20 min before being stored in 100% ethanol. Critical point drying was performed using a critical point dryer (Leica EM CPD300, Wetzlar, Germany). The samples were sputter-coated with gold and observed under a SEM (Zeiss EVO-LS10, Gottingen, Germany) at an acceleration voltage range of 5–15 kV.

### 2.13. Statistical Analysis

Each experiment was performed in triplicate, and the results were presented in the form of the mean ± standard deviation (SD). One-way Analysis of Variance (ANOVA) was employed to analyze the data using SPSS 22.0 software (SPSS Inc., Chicago, IL, USA). Differences were regarded as statistically significant when *p* < 0.05. Origin 2022 (OriginLab Corp., Northampton, MA, USA) was used for graph creation.

## 3. Results and Discussion

### 3.1. Isolation and Identification of the SSB

**Figure 1:**
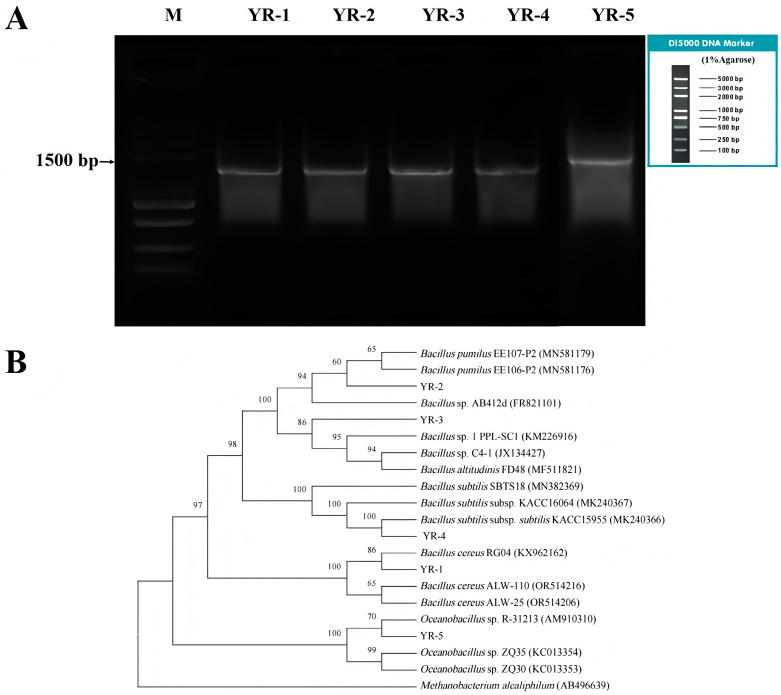
Agarose gel electrophoresis results for PCR products (**A**); phylogenetic tree based on 16S rDNA sequence homology of the five strains (**B**).

A total of eighteen strains were obtained from the spoiled CRS. Based on the different colony morphologies, five strains (YR-1, YR-2, YR-3, YR-4, and YR-5) were screened out. Using the total DNA of the five strains as a template, the bacterial 16S rDNA fragments were amplified via PCR. The agarose gel electrophoresis results (Figure 1) indicated that all five strains had clear target bands at approximately 1500 bp, demonstrating the good quality of the extracted DNA. The 16S rDNA fragments of each strain were then aligned with the most homologous strain sequences selected through NCBI and a phylogenetic tree was constructed using MEGA11. Therefore, based on the phylogenetic tree analysis (Figure 1B), the identities of each strain were determined as *B. cereus* for YR-1, *B. pumilus* for YR-2, *Bacillus altitudinis* for YR-3, *Bacillus subtilis* for YR-4, and *Oceanobacillus* sp. for YR-5. The findings indicated that during the refrigerated storage of CRS, *Bacillus* species constituted the predominant bacterial group.

In contrast to our study, Chen et al. [27] found that the *Pseudomonas* and *Shewanella* sp. were the SSB in high-moisture, high-protein foods during cold-chain distribution. This discrepancy might be attributed to the fact that the CRS in our study was subjected to low-temperature pasteurization, which effectively killed the majority of *Pseudomonas* and *Shewanella* spp., hindering their growth and proliferation. However, the low-temperature pasteurization was incapable of eradicating thermophilic bacteria and those in the *Bacillus* genus [28]. Helmond et al. [29] isolated four *Bacillus* strains from pasteurized and refrigerated ready-to-eat foods. Moreover, Pace et al. [30] showed that the main bacteria in pasteurized (80 °C, 10 min) oysters were *Bacillus*, *Corynebacterium*, *Listeria*, *Clostridium*, *Streptococcus gastricus*, and *Staphylococcus*. Additionally, Skandamis and Nychas [31] indicated that the microbes responsible for the spoilage of aquatic products were similar to those affecting meat products. Lawson et al. [32] isolated and identified the SSB in cooked pork, finding that the psychrophilic *Clostridium* genus was prevalent and caused spoilage. Furthermore, Doulgeraki et al. [33] detected the dominant bacteria in different refrigerated meat products and concluded that they were from the *Pseudomonas*, *Bacillus*, and *Staphylococcus* genera.

### 3.2. Enzymatic Characteristics of Isolated Bacteria

The enzymatic spoilage activities of the five strains are presented in Table 1.

All the strains exhibited protease, lipase, and lecithinase activities, while strains YR-2 and YR-3 lacked amylase activity. Moreover, strain YR-4 showed positive results for all enzyme activity assessments, indicating a high potential for spoilage. Larrea-Murrell et al. [34] found that the majority of *Bacillus* strains from contaminated freshwater could produce proteases, amylases, lecithinases, and lipases. Moreover, the *Bacillus* genus is the most significant bacterial source of proteases, and is capable of generating substantial quantities of neutral and alkaline proteolytic enzymes [35]. It has been observed that the proteases secreted by spoilage microorganisms during refrigeration facilitate protein degradation [36]. Consistent with the current study, various studies have confirmed that *B. cereus*, *B. subtilis*, and *B. pumilus* possess lipase and lecithinase activities [37,38,39,40]. Erfanimoghadam and Homaei [41] demonstrated that *Bacillus* species exhibited strong capabilities for starch degradation. Sam-On et al. [42] revealed that *B. pumilus* isolated from the gut of giant freshwater prawns possesses amylase activity, which was somewhat at odds with our findings. This discrepancy might be attributed to the stringent regulation of enzyme expression, resulting in the absence of one or two enzymes in certain isolated strains [43]. Previous research has reported marked phenotypic differences among strains of the same species, for instance, Caldera et al. [44] found that only 36% of *Pseudomonas fragi* strains isolated from different foods displayed protease activity. Extracellular enzymes relevant to food spoilage, particularly amylase, lecithinase, lipases, and proteases, play a crucial role in food spoilage by degrading the macromolecular components in foods and adversely affecting food quality [45]. Consequently, the five isolated *Bacillus* strains were likely to be significant contributors to the spoilage of CRS and could be considered the SSB of CRS.

### 3.3. Analysis of Spoilage Capacity of Isolated Bacteria

#### 3.3.1. Changes in TVC and TVB-N

**Figure 2:**
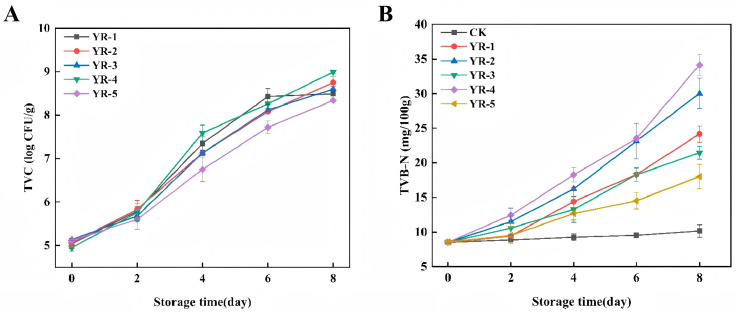
Changes in TVC (**A**) and TVB-N values (**B**) of the CRS inoculated with five isolated strains during storage at 10 °C for eight days.

The TVC is a critical indicator of food spoilage. The changes in the TVC of the CRS samples inoculated with the different strains during storage at 10 °C are depicted in Figure 2A. The TVC of all inoculated groups was essentially the same, possibly due to the time required for microorganisms to acclimate to the new environmental matrix. As the storage time was extended, all inoculated groups exhibited a growing trend, yet significant differences (*p* < 0.05) were observed among different strains, likely attributable to their distinct growth characteristics and spoilage mechanisms. The TVC of the inoculated groups reached 8.49, 8.75, 8.60, 8.99, and 8.34 log CFU/g, respectively, at the end of storage. Among these strains, YR-4 was growing fastest, while YR-2 and YR-3 showed relatively similar growth rates, and YR-5 demonstrated the slowest growth rate.

TVB-N, which is closely associated with enzymatic activity and microbial proliferation, is a critical index for evaluating the freshness of aquatic products [46]. The changes in the TVB-N values of the sterile CRS inoculated with the five strains during storage at 10 °C are depicted in Figure 2B. The initial TVB-N values of all the groups were around 8.52 mg/100 g. As the storage time increased, the TVB-N value of the CK rose in a mild manner, while that of all the inoculated groups increased rapidly, indicating an acceleration in the degree of sample spoilage. However, the TVB-N production varied among groups. At the end of storage, the TVB-N values of the YR-2 and YR-4 sample groups reached 30.02 mg/100 g and 34.13 mg/100 g, respectively, both exceeding the TVB-N limit value of 30 mg/100 g for processed animal aquatic products that is stipulated in GB-10136-2015, indicating that the samples had spoiled and were inedible. Meanwhile, the TVB-N value of the CK was merely 11.06 mg/100 g, remaining in an acceptable state, which was significantly lower than all the treatment groups (TGs) (*p* < 0.05). This suggested that these strains had strong potential to spoil CRS. Moreover, the YR-5 sample group exhibited the lowest TVB-N accumulation at 18.00 mg/100 g, indicating that the YR-5 strain decomposed proteins in CRS at the slowest rate and had the weakest capability to produce small molecular metabolites.

#### 3.3.2. Spoilage Yield Factor for SSB (Y_TVB-N/CFU_)

The spoilage capability of each strain was quantitatively characterized using the yield of spoilage metabolites per unit quantity of spoilage bacteria. As can be seen from Table 2, the magnitude of spoilage capability, in descending order, was YR-4 > YR-2 > YR-1 > YR-3 > YR-5. Among these, the spoilage metabolite yield factors of *B. subtilis* and *B. pumilus* were 6.33 and 5.83, respectively, significantly higher than that of the other groups (*p* < 0.05). This indicated that these two strains possessed the greatest potential to spoil CRS. Therefore, *B. subtilis* (YR-4) and *B. pumilus* (YR-2) were selected as the target strains in this study to investigate the antimicrobial mechanisms of the mixed bio-preservative.

### 3.4. Antimicrobial Activity of the Mixed Bio-Preservative Against B. pumilus and B. subtilis

#### 3.4.1. Diameter of Inhibition Zones

Table 3 shows the results of the measurements of the diameter of the inhibition zones for the five bio-preservatives against *B. pumilus* and *B. subtilis*. An integrated analysis revealed that, in terms of the inhibitory effect on *B. pumilus*, the five bio-preservatives ranked as follows: NS > TPs > ε-PL > GSE > RE. In the case of *B. subtilis*, TPs exhibited the most potent inhibitory action, followed by ε-PL, NS, GSE, and RE. It was noteworthy that TPs, ε-PL, and NS all exhibited favorable inhibitory effects on both target strains, with the inhibitory action intensifying as the concentration increased. Similar findings were reported by Sun et al. [47]. Specifically, they observed that NS and TPs demonstrated enhanced inhibitory effects on major spoilage (lactic acid bacteria and *Pseudomonas aeruginosa*) and pathogenic bacteria (*Staphylococcus aureus*) in pasteurized chicken sausages as the concentration increased. Therefore, TPs, ε-PL, and NS were selected for combining. The sensory properties of food are crucial determinants in food quality [48]. Therefore, while the mixed bio-preservative exerted its antibacterial effects, the impacts it had on the sensory properties of the CRS also needed to be considered. ε-PL has a long history of use as a preservative for multiple foods. It exerts excellent antibacterial effects without affecting the original flavor of the food [49]. In many traditional Japanese daily dishes, its usage concentration reaches a level of 500 ppm and it has been proposed as a preservative for rice and sushi rice, with a recommended addition level of 5–50 ppm in the United States [50]. Moreover, Li et al. [51] added 0.3% TPs to shrimp paste, and the results showed that it could effectively maintain the sensory quality of the shrimp paste when stored at 25 °C for 160 days. Dai et al. [11] also showed that the sensory scores of plant-based meat with the addition of a bio-preservative mix (2.5% TPs + 0.04% NS) were always significantly higher than those of the CK during the entire 70-day storage period. In addition, multiple studies have confirmed the salutary impacts of TPs, NS, and ε-PL in combination with other bio-preservatives on the sensory qualities of different foods [52,53,54]. Therefore, it could be inferred that the mixed bio-preservative formula in this study has potential positive effects on maintaining the sensory quality of CRS.

#### 3.4.2. MIC

The MIC is defined as the lowest antimicrobial agent concentration preventing visible bacterial growth under specified in vitro conditions and incubation times [55]. Generally, a lower MIC value indicates superior antimicrobial effectiveness. The MIC for the mixed bio-preservative against *B. pumilus* and *B. subtilis* is displayed in Table 4. No turbidity was observed in either group at a final concentration of 56.3 µg/mL of the mixed bio-preservative; hence, the MIC for both indicator strains was determined to be 56.3 µg/mL. This suggested that the mixed bio-preservative demonstrated a favorable inhibitory effect against SSB in CRS.

### 3.5. Antibacterial Mechanisms of the Mixed Bio-Preservative Against B. pumilus and B. subtilis

#### 3.5.1. Growth Curves

**Figure 3:**
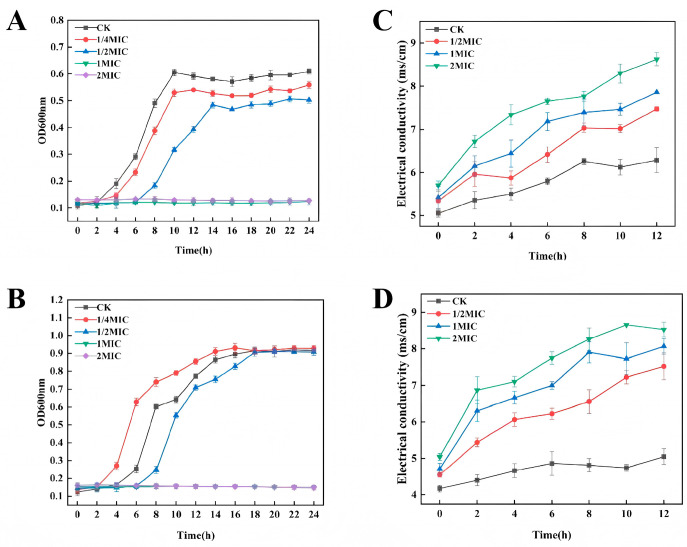
Effect of the mixed bio-preservative on the cell growth (**A**,**B**) and the electrical conductivity (**C**,**D**) of *B. pumilus* (**A**,**C**) and *B. subtilis* (**B**,**D**).

In the quantitative analysis of microorganisms, using the OD value to reflect the concentration or quantity of microorganisms is a commonly employed method. Figure 3A,B present the growth curves of *B. pumilus* and *B. subtilis*. It can be observed that the growth of both strains in the CK followed a typical “S”-shaped growth curve. At the 1/4MIC or 1/2MIC concentration of the mixed bio-preservative, the logarithmic growth phase of both strains was delayed, and bacterial growth was notably slowed, with significantly fewer bacterial counts than the CK (*p* < 0.05), indicating that low concentrations of the mixed bio-preservative reduced the vitality of the two strains and delayed their logarithmic phase. The logarithmic phase is a special period of bacterial growth and reproduction, during which the bacterial population grows rapidly and demonstrates distinct physiological features [56]. When the concentration was 1MIC or 2MIC, the growth of both strains was completely inhibited, indicating that the mixed bio-preservative significantly inhibited the growth of the two SSB and disrupted their cycle, with its antimicrobial activity depending on the concentration.

#### 3.5.2. Electrical Conductivity Analysis

The bacterial cell membrane is a protective barrier. When antimicrobial substances damage it, the barrier breaks down, causing intracellular electrolytes to leak into the cultivation medium and raising the medium’s conductivity [57]. Thus, alterations in the permeability of the bacterial cell membrane can be reflected by changes in the conductivity of the bacterial suspension. The conductivity of the *B. pumilus* suspension from the CK (Figure 3C) gradually increased during the first 8 h, reaching a peak of 6.26 ± 0.07 ms/cm before declining and stabilizing; the *B. subtilis* suspension in the CK (Figure 3D) increased slightly and maintained a relatively stable state. The conductivity increase of the CK’s bacterial suspension might be related to the use of sterile deionized water in the experiment. Bacteria lacking nutrients undergo partial lysis, leading to higher conductivity [58]. As for the TGs, the conductivities of the suspensions of both strains rose steadily with longer exposure times, and the effect intensified as the mixed bio-preservative concentration increased. The results indicated that the mixed bio-preservative altered the permeability of the cellular membranes of *B. pumilus* and *B. subtilis*.

#### 3.5.3. Nucleic Acid Leakage

**Figure 4:**
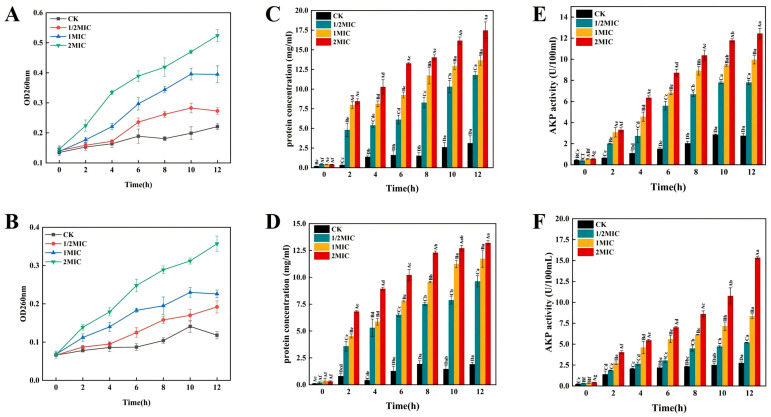
Effect of the mixed bio-preservative on nucleic acid leakage (**A**,**B**), the soluble protein content (**C**,**D**), and the AKP activity (**E**,**F**) of *B. pumilus* (**A**,**C**,**E**) and *B. subtilis* (**B**,**D**,**F**). Different uppercase letters indicate significant differences within the same treatment time among the different treatment groups (*p* < 0.05), and different lowercase letters indicate significant differences within the same treatment group at different treatment times (*p* < 0.05).

Upon cellular membrane disruption, ions such as K^+^ and PO^3-^ are initially released, followed by large molecules like DNA and RNA. Therefore, the degree of membrane damage can be judged by measuring the changes in the OD260 nm value of the bacterial suspension [59]. Figure 4A,B, respectively, present the amount of cell nucleic acid leakage after treating *B. pumilus* and *B. subtilis* with the mixed bio-preservative. As can be observed, as the incubation time increased, for both SSB cell culture solutions, the CK’s absorbance values at A260 remained relatively low throughout, while that of the TGs continued to increase and was significantly higher than the CK’s (*p* < 0.05). Moreover, the absorbance values in the 2MIC group increased at a faster speed than those in the 1MIC and 1/2MIC groups. It was clear that the mixed bio-preservative strongly affected *B. pumilus* and *B. subtilis* membranes, with the degree of damage correlating positively with its concentration.

#### 3.5.4. Bacterial Soluble Protein Analysis

A microbial biofilm’s phospholipid bilayer has many embedded protein molecules. When it is disrupted, membrane and intracellular proteins spill into the extracellular space [60]. Measuring the protein content in the bacterial suspension reveals if the bilayer is compromised. The effects of the mixed bio-preservative on the soluble protein content of *B. pumilus* and *B. subtilis* are illustrated in Figure 4C,D. As can be observed, at 12 h, the soluble protein content of *B. pumilus* treated with 1/2MIC, 1MIC, and 2MIC of the mixed bio-preservative was, respectively, 3.77, 4.36, and 5.58 times that of the CK, whereas the soluble protein content of *B. subtilis* was, respectively, 5.02, 6.11, and 6.88 times that of the CK. This indicated that a higher concentration of the mixed bio-preservative had a more significant effect on the membrane integrity, leading to cytoplasm leakage and more protein entering the medium, and thus a higher protein content. Similar results were observed by Wang et al. [61], where the bio-preservative (oregano essential oil) treatment increased the membrane permeability and leakage of intracellular proteins from *Morganella psychrotolerans*, with the protein leakage showing dose-dependency on it. Furthermore, when the conductivity of the bacterial suspension climbed rapidly, there was a concomitant rapid increase in the soluble protein content, which confirmed the damage caused by the mixed bio-preservative to the cell membranes of the two SSB [62].

#### 3.5.5. Variation in the AKP Content

Bacterial AKP lies between the cell wall and membrane, and it only leaks from the cell when the wall is damaged or its permeability changes, so its extracellular activity indirectly reflects the impact of the mixed bio-preservative on the bacterial cell wall [63]. Figure 4E,F present the levels of extracellular AKP in the *B. pumilus* and *B. subtilis* samples. As can be observed, with the prolongation of the treatment time, the AKP in the CK for the two SSB hardly leaked, while that of each TG kept increasing and was significantly higher than that of the CK (*p* < 0.05). After 12 h of treatment with the mixed bio-preservative, the AKP content of *B. pumilus* was 2.76 ± 0.23 U/100 mL in the CK, and 7.80 ± 0.22, 9.96 ± 0.40, and 12.44 ± 0.49 U/100 mL when treated with 1/2MIC, MIC, and 2MIC of the mixed bio-preservative, respectively. For *B. subtilis*, the AKP content was 2.75 ± 0.13 U/100 mL in the CK, and 5.20 ± 0.04, 8.34 ± 0.29, and 15.35 ± 0.11 U/100 mL when treated with 1/2MIC, MIC, and 2MICof the mixed bio-preservative, respectively. These results suggested that the cell walls of both strains were subject to irreversible and continuous damage under the action of the mixed bio-preservative.

#### 3.5.6. Bacterial Cell Viability Assay

**Figure 5:**
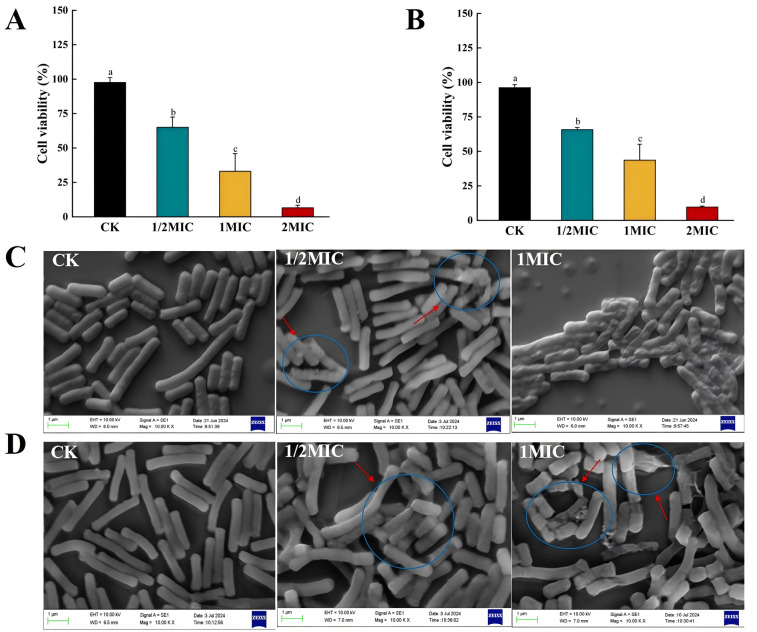
Effects of the mixed bio-preservative on the cell viability (**A**,**B**) and scanning electron microphotographs (**C**,**D**) of *B. pumilus* (**A**,**C**) and *B. subtilis* (**B**,**D**). The different letters indicate significant differences (*p* < 0.05).

The effects of the mixed bio-preservative on the cellular viability of the two SSB were detected using the CCK-8 method. When the concentrations of the mixed bio-preservative were 1/2MIC, 1MIC, and 2MIC, the cellular viability of *B. pumilus* (Figure 5A) was, respectively, 65.0%, 33.1%, and 6.6%, while that of *B. subtilis* (Figure 5B) was, respectively, 65.8%, 43.6%, and 9.6%. The differences compared to the CK were statistically significant (*p* < 0.05). The results indicated that the mixed bio-preservative diminished the cellular viability of the two SSB, inhibiting their growth. These findings were consistent with the results obtained from the growth curve measurements.

#### 3.5.7. Scanning Electron Microscopy Analysis

The SEM results for the treatment of *B. pumilus* and *B. subtilis* with the mixed bio-preservative are shown in Figure 5C,D. Untreated *B. pumilus* and *B. subtilis* exhibited plump, intact cells without deformation, whereas the bacteria treated with different concentrations of the mixed bio-preservative exhibited varying degrees of damage. The surface of the two SSB treated with 1/2MIC displayed evident layers of disruption and the appearance of holes. When treated with 1MIC, the bacterial cell membranes suffered more severe damage. This manifested as significant shriveling, rupturing, and leakage of the cell contents. Notably, the changes in cell morphology resulting from the treatment with the mixed bio-preservative might affect the contact area between viable bacterial cells and the CCK-8 solution, thus influencing the bacterial cell viability results. Moreover, the SEM findings were consistent with the conductivity, nucleic acid, and soluble protein leakage results, confirming the ability of the mixed bio-preservative to disrupt the cellular membrane. Cell structure destruction is lethal to bacteria. Their membranes, which are rich in enzyme systems, perform many metabolic functions. Once damaged, the loss of enzymes and transport proteins will affect their energy metabolism and material transport [64]. Therefore, the damage to the cellular membrane by the mixed bio-preservative was one of the critical factors leading to the death of the two SSB.

## 4. Conclusions

This study revealed the SSB in CRS and investigated the antibacterial activity and mechanisms of a mixed bio-preservative (0.025% ε-PL + 0.01% NS + 0.01% TPs) against the SSB in CRS. We isolated and identified five SSB strains from spoiled CRS, among which, *B. pumilus* and *B. subtilis* possessed the highest enzymatic activities (protease, lipase, phospholipase, and amylase activities). Moreover, these two isolates exhibited strong spoilage capabilities in CRS, which were revealed by them having the highest Y_TVB-N/CFU_. The mixed bio-preservative presented significant antibacterial activity against *B. pumilus* and *B. subtilis*, with an MIC of 56.3 µg/mL for both strains. The mixed bio-preservative effectively inhibited the growth of these two strains, disrupted the structure and function of the cell walls and membranes of the spoilage bacteria, induced the leakage of intracellular contents, and impacted the normal energy metabolism of bacterial cells, and therefore led to cell death. The SEM results further confirmed the damage to the bacterial cell membrane caused by the mixed bio-preservative. The findings of this study provide a theoretical basis for the application of the mixed bio-preservative in the preservation of CRS.

## Figures and Tables

**Table 1:** Enzymatic activity of five strains.

Strain	Species	Enzymatic Spoilage Potential			
		Protease	Lecithinase	Amylase	Lipase
YR-1	*B*. *cereus*	±	+	±	+
YR-2	*B*. *pumilus*	+	+	−	+
YR-3	*B*. *altitudinis*	+	+	−	+
YR-4	*B*. *subtilis*	+	+	+	+
YR-5	*Oceanobacillus* sp.	±	+	+	+

Note: +: positive result; ±: weak positive result; and −: negative result.

**Table 2:** Yield factors of Y_TVB-N/CFU_ of five strains.

Strain	TVC (log_10_CFU/g)		TVB-N (mg/100 g)		Y_TVB-N/CFU_
	Initial	Final	Initial	Final	
YR-1	5.04 ± 0.12 ^ab^	8.49 ± 0.05 ^c^	8.48 ± 0.05 ^b^	24.15 ± 1.16 ^c^	4.54
YR-2	5.06 ± 0.11 ^ab^	8.75 ± 0.13 ^b^	8.52 ± 0.02 ^ab^	30.02 ± 2.20 ^b^	5.83
YR-3	5.14 ± 0.02 ^a^	8.60 ± 0.06 ^c^	8.52 ± 0.05 ^ab^	21.45 ± 0.94 ^c^	3.74
YR-4	4.95 ± 0.07 ^b^	8.99 ± 0.04 ^a^	8.54 ± 0.03 ^ab^	34.13 ± 1.48 ^a^	6.33
YR-5	5.12 ± 0.02 ^a^	8.34 ± 0.02 ^d^	8.56 ± 0.02 ^a^	18.00 ± 1.77 ^d^	2.93

Note: Data are expressed as the mean ± SD of triplicate assays. Different letters indicate that there are significant differences among different strain inoculation groups at the same time (*p* < 0.05).

**Table 3:** Diameter of inhibition zones of each of five bio-preservatives alone against *B. pumilus* and *B. subtilis*.

Bio-Preservative	Concentration	Inhibition Zone (mm)	
		*B. pumilus*	*B. subtilis*
TPs	0.1%	-	11.51 ± 0.13 ^a^
	0.2%	10.87 ± 0.62 ^a^	13.65 ± 0.18 ^a^
	0.3%	12.53 ± 0.43 ^a^	14.28 ± 0.28 ^a^
	0.4%	13.88 ± 1.49 ^a^	14.95 ± 0.06 ^a^
	0.5%	13.97 ± 0.63 ^a^	16.07 ± 1.54 ^a^
NS	0.1%	8.22 ± 0.07	8.53 ± 0.66 ^b^
	0.2%	12.10 ± 0.90 ^a^	10.98 ± 0.61 ^b^
	0.3%	13.62 ± 0.05 ^a^	11.04 ± 0.58 ^c^
	0.4%	13.92 ± 0.86 ^a^	13.29 ± 0.82 ^b^
	0.5%	15.21 ± 0.73 ^a^	13.63 ± 1.01 ^b^
RE	0.1%	-	-
	0.2%	-	-
	0.3%	-	-
	0.4%	-	-
	0.5%	-	-
GSE	0.1%	-	9.00 ± 0.27 ^b^
	0.2%	-	10.57 ± 1.19 ^b^
	0.3%	-	10.67 ± 0.17 ^c^
	0.4%	10.10 ± 1.34 ^b^	10.78 ± 0.48 ^c^
	0.5%	11.03 ± 2.27 ^b^	11.41 ± 0.59 ^c^
ε-PL	0.1%	7.87 ± 0.36	11.17 ± 1.21 ^a^
	0.2%	10.94 ± 0.41 ^a^	11.88 ± 0.27 ^b^
	0.3%	12.87 ± 1.11 ^a^	13.59 ± 0.57 ^b^
	0.4%	11.52 ± 1.82 ^ab^	14.19 ± 0.02 ^ab^
	0.5%	13.87 ± 0.66 ^a^	16.07 ± 1.54 ^a^

Note: The absence of significant inhibition zones is denoted by a dash (-). Data are expressed as the mean ± SD of triplicate assays. Different letters indicate significant differences (*p* < 0.05) among the various treatment groups at the same concentration.

**Table 4:** MIC of the mixed bio-preservative.

Strain	(0.025% ε-PL + 0.01% NS + 0.01% TPs) (µg/mL)									
	450	225	113	56.3	28.1	14.1	7.03	3.52	Positive Control	Negative Control
YR-2	−	−	−	−	+	+	+	+	+	−
YR-4	−	−	−	−	+	+	+	+	+	−

Note: + means bacterial growth and − means no bacterial growth.

## Data Availability

The original contributions presented in the study are included in the article; further inquiries can be directed to the corresponding author.
